# Addressing Knee Osteoarthritis Pathology Through Platelet-Rich Plasma Treatment: A Comprehensive Review

**DOI:** 10.1155/2024/6551525

**Published:** 2024-11-19

**Authors:** Silviu Valentin Vlad, Timea Claudia Ghitea, Felicia Manole, Alexandru–Stefan Nutiu, Alex Octavian Lupsa, Nicu Adrian Ghiurau, Florin Nicolae Blaga

**Affiliations:** ^1^Traumatology and Orthopedics Department, County Clinical Emergency Hospital of Oradea, 65 Gheorghe Doja Street, Oradea 410169, Romania; ^2^Department of Surgical Specialties, Faculty of Medicine and Pharmacy, University of Oradea, 10 1st Decembrie Street, Oradea 410073, Romania; ^3^Pharmacy Department, Faculty of Medicine and Pharmacy, University of Oradea, 10 1st Decembrie Street, Oradea 410073, Romania

**Keywords:** intra-articular, knee, orthopedic, osteoarthritis, platelet-rich plasma, treatment

## Abstract

Platelet-rich plasma (PRP) is gaining popularity across various medical fields, including orthopedics, for its potential in tissue regeneration and wound healing. As intra-articular treatments evolve, PRP has emerged as a promising option for managing knee osteoarthritis, meniscus, and ligament injuries. This review aims to provide an update on the current applications of PRP in treating knee osteoarthritis and its clinical implications in orthopedic and sports medicine. We reviewed 180 eligible studies, and our findings suggest that PRP injections significantly improve knee joint function compared to alternative treatments. The use of PRP across various medical fields has been growing in popularity recently. PRP is a biological product derived from the plasma portion of a patient's own blood, containing a higher concentration of platelets than normal. Its potential for tissue regeneration and wound healing has drawn significant attention from orthopedic surgeons, especially as intra-articular treatment options continue to evolve. The benefits of PRP in treating various osteoarticular conditions have sparked considerable interest within the orthopedic community, particularly for managing knee osteoarthritis, meniscus tears, and ligament injuries. This review aims to provide an updated overview of the current applications of PRP in the treatment of knee osteoarthritis and to offer clinical insights into its use in orthopedic and sports medicine practices. We reviewed 180 relevant titles and abstracts that met the inclusion criteria. Compared to other treatment options, PRP injections significantly enhance knee joint function.

## 1. Introduction

Osteoarthritis (OA) is a degenerative joint disease that predominantly affects women over the age of 65, impacting over 300 million people worldwide. For these women, the knee is the most commonly affected joint, accounting for 89% of all OA cases. Although life expectancy has increased, issues like obesity are on the rise, along with a growing number of sports-related injuries, leading to a higher prevalence of knee-related pathologies [1]. Contributing factors such as hormonal imbalances [2], improper use of medications [3], insufficient rehabilitation after trauma or surgery [4], and a lack of awareness about these conditions are all closely linked [4].

OA is a leading cause of disability, affecting over 300 million people worldwide, with the knee being the most commonly affected joint. The increasing prevalence of knee osteoarthritis (KOA), particularly among older adults and those with obesity, poses significant challenges to the healthcare system. Conventional treatments, such as NSAIDs and corticosteroids, provide only temporary relief and may have adverse effects, while surgical options are often reserved for advanced cases. Platelet-rich plasma (PRP) has emerged as a promising biological therapy due to its potential to enhance tissue regeneration and modulate inflammation. This review aims to explore the role of PRP in managing KOA, examining its efficacy, safety, and long-term outcomes [5–7].

Despite being extensively studied and highly significant in modern medicine [8, 9], the complexities of the knee joint and the pathologies it can develop are still not fully understood [10, 11].

The use of hyaluronic acid (HA), especially when combined with treatments like extracorporeal shock wave therapy [12], has shown significantly better results compared to treatments without HA [13].

This review aims to address the most common osteoarticular pathologies, focusing on three prevalent conditions, and to provide a detailed overview of the current use of PRP, its physiological mechanisms, and the benefits it offers.

## 2. Materials and Methods

### 2.1. Search Strategy

This review follows a comprehensive review methodology, aiming to provide an in-depth overview of the use of PRP in KOA treatment without the constraints of systematic review protocols. Although we employed a structured search strategy and selection criteria similar to systematic reviews, our focus is broader, encompassing various aspects of PRP application, including clinical outcomes, safety, and potential molecular mechanisms. We performed a review to evaluate the properties and efficacy of PRP as an intra-articular treatment for knee-related conditions, with a primary focus on KOA.

This narrative review was conducted by searching three major databases: Google Scholar, PubMed, and MDPI. To locate the most relevant studies, we used a combination of keywords such as “knee osteoarthritis,” “PRP treatment,” “osteoarthritis,” “knee pathology,” and “platelet-rich plasma.” The search covered literature published between 1974 and 2023. The search strategy was specifically designed to find studies exploring the anti-inflammatory, analgesic, and antiaging effects of PRP in the treatment of knee pathologies, with an emphasis on KOA. This section may include subheadings and should provide a clear and concise presentation of the experimental results, their interpretation, and the conclusions that can be drawn from the findings.

### 2.2. Study Selection and Eligibility Criteria

All the titles, selected abstracts, and full-text publications identified through the electronic search were independently reviewed by at least four reviewers to ensure the reliability and accuracy of the selection process. We specifically included studies that contained the relevant keywords mentioned earlier, such as “knee osteoarthritis,” “PRP treatment,” “osteoarthritis,” “knee pathology,” and “platelet-rich plasma.” Although there were no restrictions on the publication year, the majority of the studies included in our review were published after 2019, reflecting the most recent advancements and findings in this area of research.

Our initial database search yielded a total of 236 records. After removing 20 duplicate entries, we were left with 216 unique articles. We then screened these articles for relevance, eliminating 36 studies that did not meet our inclusion criteria due to their focus or scope being unrelated to the topic at hand. This thorough vetting process left us with 180 titles and abstracts that were deemed eligible for a more detailed review. After a comprehensive evaluation of these 180 studies, we found that 105 of them met the rigorous criteria established for this review. These selected references were subsequently included in the final analysis, providing a robust and comprehensive foundation for our assessment of the use of PRP in the treatment of KOA.

#### 2.2.1. Inclusion Criteria

For women over the age of 65, this review included a wide range of knee conditions that are often of concern at this stage of life. These conditions encompassed both primary and secondary OA of the knee, which are common degenerative joint diseases that many older women experience. It also included other issues such as osteochondritis dissecans, which can lead to joint instability, and degenerative meniscal lesions, as well as injuries to the meniscus or ligaments. The review also considered studies where patients, possibly women like me, underwent various knee surgeries such as meniscectomy, meniscus-sparing surgery, meniscus replacement, ligament reconstruction, and knee osteotomy.

#### 2.2.2. Review Focus

The review was particularly focused on assessing the treatment options for KOA, a condition that significantly affects older women like me, along with other knee problems such as osteochondritis and injuries to the meniscus and ligaments. It evaluated the effectiveness of modern treatment methods, ranging from conservative options and minimally invasive procedures to more involved surgical approaches. The review covered a variety of therapies, including PRP injections, the use of synovial mesenchymal stem cells, HA treatments, extracorporeal shock wave therapy, and high-intensity laser treatments.

#### 2.2.3. Exclusion Criteria

**Figure 1:**
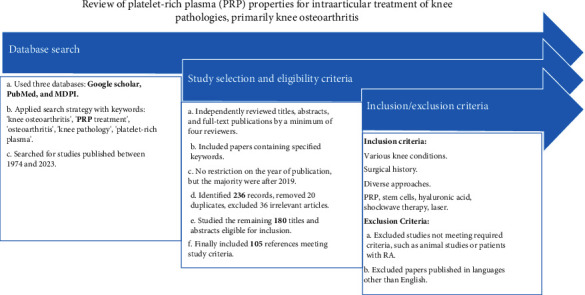
Flowchart.

Studies that did not meet the specific requirements, such as those conducted on animals or involving patients with rheumatoid arthritis, were excluded from the review. Additionally, research papers that were not published in English were not considered, as they might not have been accessible for older women like me who speak only English. Any disagreements among the reviewers regarding which studies should be included or excluded were resolved through a consensus process to ensure the reliability of the review. This thorough screening is represented in the flowchart in Figure 1.

## 3. KOA

OA is a common and challenging condition that affects millions of people around the world, especially those of us over the age of 65. It is estimated that about 300 million people globally suffer from this painful joint disease, with the knee being the most frequently affected joint. In fact, KOA accounts for a staggering 89% of all OA cases. This puts a significant burden not only on our daily lives but also on the healthcare system, as managing this condition requires ongoing care and treatment [14, 15].

There are several factors that contribute to the development of OA, particularly as we get older. Age is, of course, a major factor; our joints naturally experience more wear and tear over the years. For many women like me, maintaining a healthy weight becomes more challenging as we age, and excess weight can place additional strain on our knees, accelerating the breakdown of the joint cartilage. Additionally, some of us are more prone to developing OA due to genetic factors that we have inherited. It is not just age and genetics, though mechanical stress from everyday activities, past injuries, or even long-term physical work can also take a toll on our joints, contributing to the onset and progression of this disease [16].

The progression of KOA does not happen overnight; it typically unfolds in three stages. In the beginning, there is a process called proteolytic degradation, where enzymes start to break down the proteins in the cartilage. This is the smooth, protective tissue that covers the ends of the bones in our joints. As the condition progresses, we experience fibrillation, where the cartilage surface becomes rough and frayed. This means it is no longer providing the cushioning and protection our knees need. Eventually, in the final stage, the cartilage thins out and wears away even more, leading to increased pain, stiffness, and a significant reduction in mobility. This is often when we start noticing that it is becoming more difficult to carry out daily activities that we used to take for granted [17, 18].

**Figure 2:**
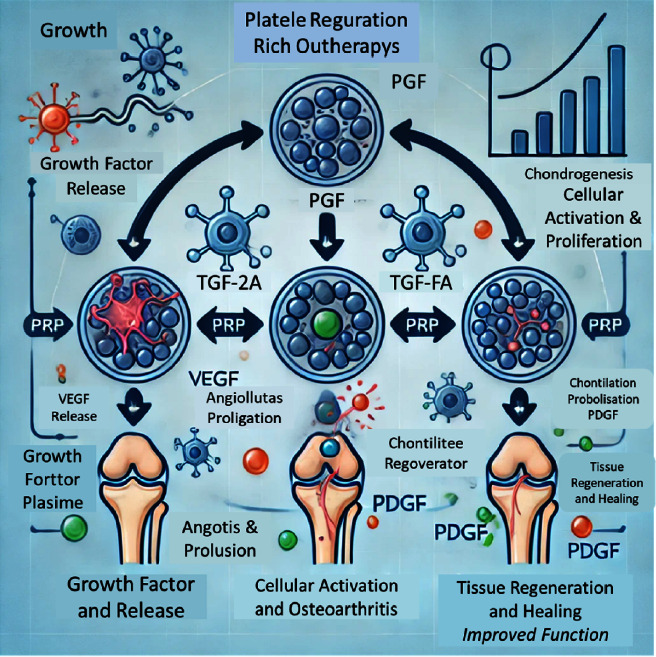
Stages of KOA.

Understanding how OA progresses is crucial for us to seek the right treatments and take steps to manage the condition effectively, aiming to maintain our mobility and quality of life as we age [19] (Figure 2).

In the third phase of OA progression, the condition intensifies as the cartilage continues to break down, leading to significant structural and functional changes in the joint. During this phase, the degradation of essential components such as collagen and proteoglycans becomes more pronounced. Collagen, a critical protein that provides strength and structure to the cartilage, begins to deteriorate, compromising the integrity of the joint tissue. Simultaneously, proteoglycans, which help maintain the cartilage's elasticity and ability to absorb shock, also undergo degradation, further weakening the cartilage's capacity to protect the bones.

Moreover, this phase is marked by a substantial increase in the production of pro-inflammatory enzymes. These enzymes, including matrix metalloproteinases (MMPs) and aggrecanases, are responsible for breaking down cartilage components and are upregulated in response to ongoing inflammation. Their heightened activity not only accelerates the degradation process but also contributes to the chronic inflammation that characterizes advanced OA.

**Figure 3:**
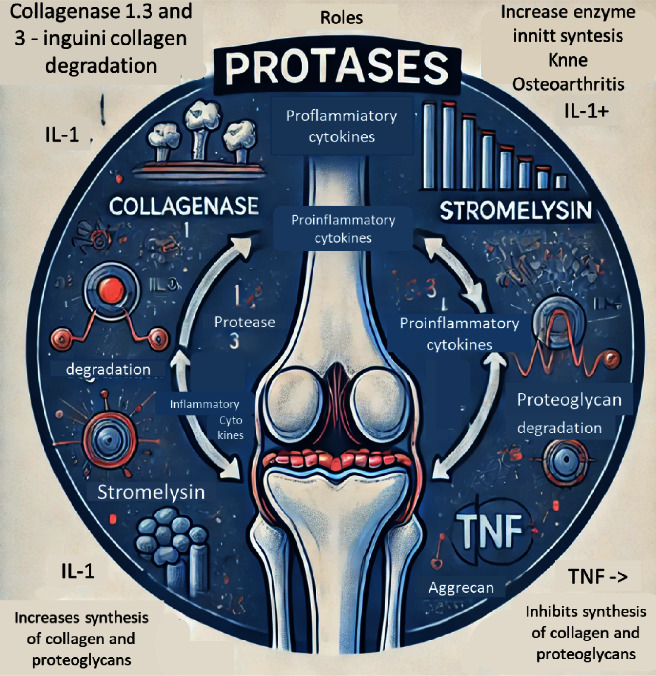
Third-stage KOA physiopathological pathways.

Additionally, the synthesis of matrix constituents—critical substances such as collagen and glycosaminoglycans that are necessary for the maintenance and repair of healthy cartilage—is significantly inhibited. This impaired regenerative capacity means that the cartilage is unable to repair itself effectively, leading to a vicious cycle where the breakdown of cartilage outpaces the body's ability to rebuild it. Consequently, this phase of the disease results in further thinning of the cartilage, increased joint pain and stiffness, and a noticeable decline in joint function, greatly impacting the quality of life for those affected [18, 20–24] (Figure 3).

Age is one of the most significant risk factors contributing to the development and progression of OA, particularly due to its impact on the structural integrity of the cartilage's extracellular matrix (ECM) [25]. As individuals age, the ECM, which is crucial for maintaining the mechanical properties of cartilage, undergoes various changes that compromise its ability to withstand mechanical stress. These alterations include the loss of proteoglycans and changes in collagen structure, leading to a reduction in cartilage elasticity and resilience.

OA is not limited to the degeneration of articular cartilage alone but extends to a broader array of joint tissues, including the subchondral bone, synovium, and connective tissues. This widespread degeneration results in a disruption of normal joint metabolism, further exacerbating the disease process [26, 27]. The pathological changes in OA are both structural and molecular, affecting the entire joint as a unit rather than just individual components. This holistic involvement of the joint is what makes OA such a challenging condition to manage [28].

The hallmark features of OA include cartilage erosion, which is the progressive thinning and degradation of the cartilage layer, and significant tissue loss within the joint. Additionally, the subchondral bone, which lies just beneath the cartilage, often responds to the loss of cartilage by forming cysts and developing sclerosis. Osteophytes, commonly known as bone spurs, frequently form along the joint margins as a result of the body's attempt to stabilize the deteriorating joint [29]. These bony outgrowths can contribute to pain and further restrict joint mobility.

**Figure 4:**
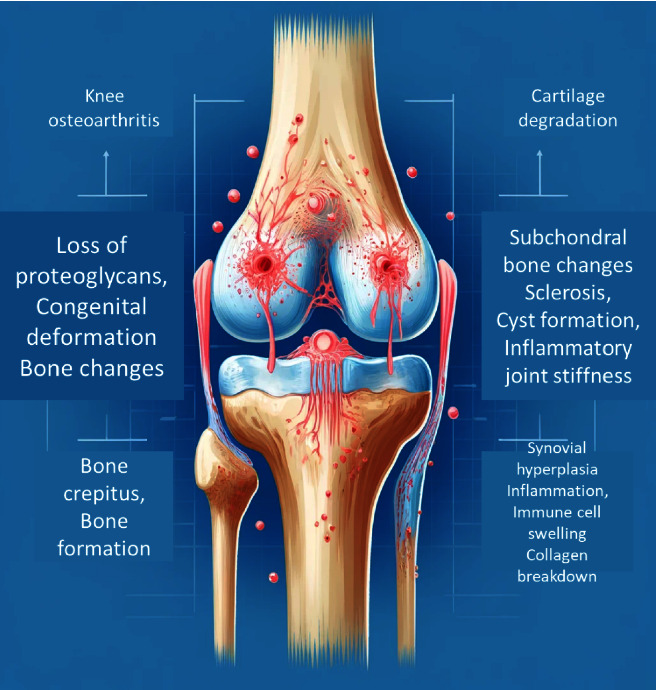
“Cartilage degradation,” “Subchondral bone changes,” and “Synovial inflammation.” Each stage should include key elements: (1) cartilage degradation: loss of proteoglycans, collagen breakdown, and chondrocyte apoptosis. (2) Subchondral bone changes: bone sclerosis, osteophyte formation, and bone marrow lesions. (3) Synovial.

The clinical manifestations of KOA are multifaceted and typically include chronic pain, stiffness, swelling, and a marked reduction in joint function. These symptoms significantly impact the quality of life, often leading to disability in severe cases. Radiographic imaging, such as X-rays, remains a primary diagnostic tool, revealing joint space narrowing, osteophyte formation, and subchondral sclerosis. Magnetic resonance imaging (MRI) and computed tomography (CT) scans provide more detailed assessments, allowing for the evaluation of soft tissue structures and early cartilage changes that are not visible on standard radiographs [30] (refer to Figure 4 for visual representation).

The degeneration of cartilage integrity is primarily due to the breakdown of the ECM and changes in chondrocyte function. Chondrocytes, the cells responsible for maintaining healthy cartilage, undergo phenotypic changes in OA, shifting from a homeostatic to a catabolic state. This leads to increased production of degradative enzymes, such as MMPs, which further degrade the cartilage matrix. As the cartilage loses its proteoglycan content, it becomes more permeable and starts to accumulate water. This change in composition, combined with a reduction in collagen network integrity, results in a significant decrease in cartilage stiffness and resilience.

Subchondral bone cysts, which form as a response to the altered mechanical loading of the joint, and osteophytes are common pathological features of advanced KOA [29]. These structural changes contribute to the clinical symptoms and progressive nature of the disease, underscoring the need for comprehensive diagnostic approaches and targeted treatment strategies.

In summary, the primary clinical symptoms associated with KOA include persistent pain, joint stiffness, functional impairment, and swelling. Accurate diagnosis typically involves a combination of clinical assessment and imaging modalities, such as radiography, MRI, or CT scans, to evaluate the extent of joint damage and guide treatment decisions [30]. Understanding the complex interplay of structural and molecular changes in OA is essential for developing effective therapeutic interventions aimed at halting or slowing the disease progression.

This diagram shows the crucial structural and molecular changes that happen in KOA, a condition that many of us women over the age of 65 are all too familiar with. The progression is divided into three stages, each worsening the condition over time. The first stage, known as cartilage degradation, is when the protective cartilage in our knees begins to break down. This process starts with the loss of essential components called proteoglycans and the breakdown of collagen, which normally helps keep our cartilage strong and flexible. As these substances diminish, the cartilage loses its elasticity and resilience, making it less able to cushion our joints. Additionally, the cells that maintain the cartilage, called chondrocytes, begin to die off, further worsening the situation and leading to more cartilage damage.

In the second stage, subchondral bone changes, and the underlying bone beneath the cartilage starts to react to the increased stress from the deteriorating cartilage. The bone becomes denser and harder, a process known as bone sclerosis. Alongside this, osteophytes, or bone spurs, form around the edges of the joint. These bony growths can cause significant pain and limit our ability to move freely. Furthermore, lesions can form in the bone marrow, adding to the pain and further contributing to the progression of the disease. The final stage, synovial inflammation, involves changes in the soft tissue lining the joint, known as the synovium. As the disease progresses, the synovial membrane thickens due to an increase in the number of synovial cells, a condition called synovial hyperplasia. This thickening leads to inflammation, which is made worse by elevated levels of inflammatory substances called cytokines. The result is joint swelling, increased pain, and even more difficulty moving the joint, making everyday activities more challenging. This diagram effectively captures the complex series of events that occur in KOA, illustrating how the disease progresses from cartilage breakdown to bone changes and inflammation. Understanding these stages helps explain why KOA can be so debilitating and highlights the importance of early intervention and treatment to slow down these changes and maintain joint health for as long as possible. Ligament and meniscus injuries: In younger patients (25–50 years old), ligament and meniscus injuries are prevalent, increasing the risk of OA in the long term [31, 32]. Contrary to previous beliefs, these injuries may not always result from traumatic events, suggesting a connection with OA development [33–35]. Strategies to prevent and delay OA onset are crucial, especially in this demographic [36].

## 4. Pharmacological Treatment of KOA

As a woman over the age of 65, I know that treatments for KOA often focus on short-term relief, but considering how this condition affects our daily lives over the long haul, it is vital to seek solutions that offer lasting results [37, 38]. Simple, cost-effective measures like making lifestyle changes and losing weight are often recommended but, unfortunately, they are not always emphasized or followed as they should be, despite their potential benefits [39, 40]. When we first start treatment, the goal is usually to relieve the pain and discomfort using a mix of non-drug and drug therapies [41]. However, these methods, while helpful initially, often only provide temporary relief [42]. For example, corticosteroid injections, which are commonly prescribed, may give us some short-term pain relief, but they can also cause further damage to the cartilage, potentially making the situation worse over time [43, 44]. While these treatments might seem like a quick fix, the benefits do not last long, and the side effects—especially the risk of cartilage degeneration from repeated steroid injections—can actually lead to even more pain and stiffness in the long run. This is why it is so important for us to consider treatment options that not only help us feel better today but also protect our knees for the future [45].

### 4.1. PRP

As a woman over 65, I have learned that platelets, also known as thrombocytes, originate from the bone marrow and play a crucial role in our body's healing processes. These small, disk-shaped cells are the tiniest in our bloodstream, measuring around 2 μm in diameter, and they are present in healthy individuals at concentrations ranging from 150,000 to 400,000 platelets per microliter of blood [46–48]. The primary function of platelets is to help our blood clot when we are injured. They do this through three main actions: adhesion, activation, and aggregation. When we get a cut or any vascular injury, platelets spring into action, sticking to the damaged area, activating, and releasing special substances from their granules that trigger the clotting process [49].

Although we used to think of platelets mainly as agents of blood clotting, recent research has shown they have much more to offer, especially for people like me who might be dealing with conditions like OA. Now we know that platelets also play significant roles in reducing inflammation, supporting the movement and growth of stem cells, and encouraging the formation of new blood vessels—all thanks to the growth factors and cytokines they release [50].

When platelets are used in PRP treatments, they are activated, releasing these valuable growth factors into the surrounding tissues. The ones that are particularly interesting for us include the vascular endothelial growth factor (VEGF), which promotes blood vessel growth; the transforming growth factor-beta (TGF-β1), which helps regulate tissue repair and balance; the platelet-derived epidermal growth factor (PDEGF), which also aids in healing and angiogenesis; the insulin-like growth factor (IGF), which stimulates cartilage and bone growth; and the basic fibroblast growth factor (b-FGF or FGF-2), which supports the production of collagen and promotes cell growth [31]. Understanding these functions helps explain why PRP can be so beneficial for healing and tissue regeneration. It is not just about stopping bleeding anymore; it is about harnessing these powerful growth factors to improve our quality of life. Whether it is promoting the growth of new blood vessels, supporting cartilage and bone health, or helping our tissues repair themselves, these growth factors are key players in how our bodies recover and stay healthy, even as we age [48, 51, 52].

**Figure 5:**
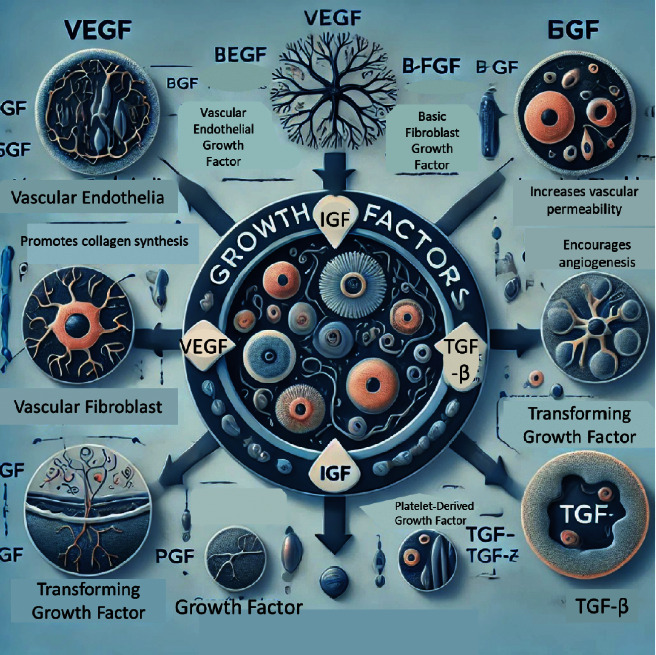
The functions and characteristics of the five key growth factors: basic fibroblast growth factor (b-FGF), vascular endothelial growth factor (VEGF), platelet-derived epidermal growth factor (PDEGF), transformative growth factor beta (TGF-*β*1), and insulin-like growth factor (IGF).

The diagram should include five growth factors: (1) b-FGF, (2) VEGF, (3) PDEGF, (4) TGF-β1, and (5) IGF. For each growth factor, list its main functions: For b-FGF, include functions like activating KGF production, promoting collagen synthesis, and stimulating cell proliferation. For VEGF, include promoting angiogenesis and wound healing. For PDEGF, include stimulating angiogenesis and wound healing. For TGF-β1, include regulating the balance between fibrosis and myocyte regeneration. For IGF, include stimulating cartilage and bone growth. Use a clean and professional design with a blue and white color scheme (Figure 5).

### 4.2. PRP Preparation Interaction With Molecular Pathways

PRP is prepared through differential centrifugation, which separates blood into different layers and can be done using one of two methods: the open method, which carries a risk of contamination [53–56] and the closed method, which utilizes anticoagulants to reduce this risk [57]. After centrifugation, PRP typically appears as a three-layered composition, and there is ongoing discussion in the scientific community regarding whether to include leukocytes in the PRP formulation [58–60]. Additionally, debates continue on the best way to activate PRP; for instance, substances like calcium gluconate are sometimes used to trigger localized clotting effects [61, 62].

The optimal concentration of PRP remains a topic of contention. While current devices can concentrate platelets to levels that are 2–5 times higher than the baseline, concentrations exceeding 2.5 times the baseline may potentially inhibit the desired therapeutic effects [63–65]. Once administered, the growth factors released by PRP can have effects lasting up to a year. However, due to the relatively short lifespan of platelets, repeated treatments at close intervals may be necessary to maintain therapeutic benefits [61].

Researchers are also investigating the use of different carriers, such as gelatin hydrogels, hydroxyapatite, and chitosan PRP hybrids, to improve the stability and efficiency of growth factors, with promising results observed in animal studies [66, 67].

PRP achieves its therapeutic benefits by releasing a variety of growth factors and cytokines that interact with several molecular pathways in the body. For example, VEGF and b-FGF stimulate the formation of new blood vessels, while TGF-β1 plays a role in controlling inflammation and fibrosis. IGF is involved in cartilage regeneration by promoting chondrocyte proliferation and matrix synthesis. Together, these interactions enhance tissue repair and may help slow the progression of OA.

## 5. Benefits of PRP Treatment in Knee Pathology

As a woman over the age of 65, it is encouraging to see that there is growing interest from orthopedic and sports medicine professionals in using PRP treatments to address issues with ligaments, tendons, and bones [66]. This surge in interest means there are more options for us when it comes to treating joint and soft tissue problems. The growth factors released by PRP play a significant role in healing by helping the body recruit cells to the injury site, promote the formation of new blood vessels (angiogenesis), and encourage cell growth [68, 69]. This not only reduces inflammation but also supports the overall healing process, which is particularly important for those of us dealing with chronic joint issues.

PRP helps improve the metabolic functions of damaged tissues by sending out signals that can stimulate the growth and proliferation of stem cells, which in turn can enhance cartilage regeneration [55, 70, 71]. This means it has the potential to support the repair of our joints in a way that conventional treatments may not be able to.

The growth factors found in platelets can contribute to cartilage repair by aiding protein production, cell growth, and cell migration, as well as helping to synthesize the entire cartilage matrix [72]. When applied to chondrocytes (the cells in cartilage), PRP can kick-start the body's natural healing processes while simultaneously controlling inflammation, which is crucial for those of us suffering from OA.

For joints affected by OA, PRP can influence not only the cartilage cells but also the surrounding synovial and endothelial cells, as well as the bone elements within the joint [73]. By reducing inflammation and encouraging the formation of new blood vessels, PRP can help slow the progression of the disease and support the joint's overall health [51].

Given its ability to promote the growth of various cells, such as tenocytes, osteoblasts, and mesenchymal stem cells, PRP could potentially serve as a primary pain-relieving treatment [74, 75]. By encouraging cartilage growth and affecting the entire joint complex, PRP injections can offer short-term relief from OA symptoms and may even delay the need for more invasive procedures like knee replacement surgery [76]. Although the results of some studies remain inconclusive, PRP treatments have generally shown better outcomes compared to other treatments like HA or placebo, at all stages of KOA [77]. PRP seems to offer better joint function and longer-lasting symptom relief, with patients experiencing improved outcomes at 3, 6, and even 12 months compared to those who received placebo, steroids, or HA injections [78–80]. Additionally, the WOMAC score, which measures pain, stiffness, and physical function, tends to be lower (indicating better results) in those receiving PRP than in those treated with HA or corticosteroids [81].

While most studies suggest that PRP is a relatively safe treatment with good outcomes, it is important to be aware that there are still some drawbacks and potential risks associated with it. However, knowing that PRP is generally considered safe and can offer promising results gives hope to many of us looking for effective ways to manage our joint pain and improve our quality of life [31] (Table 1).

Jang and colleagues conducted a study involving 65 patients with KOA who received a single intra-articular PRP injection. Most patients reported clinical improvement after 6 months; however, the benefits tended to diminish by the one-year follow-up [82]. In a separate study, Torrero and his team examined 30 patients with knee chondropathy, ranging from Outerbridge Grades I to III. They observed positive clinical outcomes at the 6-month mark following a single intra-articular PRP injection [83].

Hart et al. conducted a year-long clinical trial on 51 patients with chondromalacia Grades II and III, administering a total of nine autologous PRP injections. The results showed significant clinical improvement throughout the study period [84]. Patel and colleagues carried out a randomized controlled trial on 78 patients with bilateral KOA, totaling 156 knees. Participants receiving either a single PRP injection or two PRP injections showed greater clinical improvement compared to those who received saline injections. Interestingly, a single dose of PRP with filtered white blood cells was just as effective as two injections in improving clinical outcomes [85].

Filardo and his team compared two different PRP preparation techniques in a study involving 144 patients with OA and degenerative cartilage lesions. Both groups experienced significant clinical improvements at the one-year follow-up. Notably, patients treated with the single-spinning method reported less pain and swelling compared to those who received the other preparation method [76].

While PRP therapy shows promise in reducing symptoms and improving function in KOA, it is not without limitations. The variability in PRP preparation methods and platelet concentrations can lead to inconsistent outcomes. Potential risks include local reactions such as pain, swelling, and, in rare cases, infection. Moreover, the long-term stability of PRP effects remains under investigation, with some studies reporting diminishing benefits after 1 year of treatment. Future research should focus on standardizing PRP preparation and administration protocols to ensure consistent clinical outcomes.

Bansal and colleagues conducted a study over three years, involving 150 randomly selected patients, with 75 receiving PRP treatment and the other 75 treated with HA. Both groups experienced significant improvement at the 1-month mark. However, the benefits of PRP were sustained for up to a year, whereas the HA group saw a gradual decline in effectiveness during follow-up assessments at 3, 6, 9, and 12 months [64].

In the context of total knee arthroplasty (TKA), the use of PRP has been reported in various studies, highlighting its effects as summarized in Table 2.

## 6. Discussion

Clinical studies have presented strong evidence indicating the potential effectiveness of PRP therapy in treating degenerative joint diseases of the knee [30]. Compared to other treatment options, a 2017 study revealed that PRP injections significantly enhance knee joint function and provide superior pain relief [92]. The straightforward preparation and administration process, along with the minimal need for specialized medical equipment, make PRP a practical option for routine use in orthopedic and sports medicine settings [93].

However, according to Tey in 2022, the varying methods of PRP preparation, which often depend on physician preference, create challenges in developing standardized guidelines that can be broadly applied to all patients [94]. Therefore, further research is needed to refine and establish specific protocols that take into account the diverse ways PRP is prepared. Although current studies show promising results, the lack of standardized guidelines highlights the need for more research to create comprehensive protocols that can be universally adopted [95].

PRP therapy's flexibility in adapting to various clinical scenarios, combined with its ease of use and favorable safety profile, makes it a promising option for managing KOA [96, 97]. Although definitive guidelines are still lacking, recent studies' positive outcomes contribute to the growing understanding of PRP's potential benefits for knee joint health [98], and the importance of vitamin D in reducing the inflammatory process [99, 100]. As research continues to progress, it is expected that a more detailed and precise framework for PRP application will be developed, providing healthcare providers with clearer insights into how best to utilize this treatment for better patient outcomes.

Our understanding of knee pathologies is evolving, especially regarding the complex interactions between aging, trauma, and the development of OA. This evolving knowledge encourages the exploration of various preventive measures and calls for a reexamination of traditional views on the causes of OA. Ongoing research and refinement of these concepts are crucial for developing effective PRP-based interventions to reduce the impact of OA on individuals and healthcare systems. Recent studies that highlight these aspects are essential for advancing our understanding and improving treatment strategies, thereby establishing a solid foundation for using PRP as a viable intervention for managing knee conditions. This continuous exploration is vital for tailoring PRP treatments to address the specific factors contributing to knee pathologies, providing targeted and innovative solutions to enhance patient outcomes.

## 7. Conclusions

In conclusion, PRP therapy offers a promising alternative for managing KOA, demonstrating benefits in reducing pain and improving joint function. However, variability in preparation methods and limited long-term data warrant caution. Future research should focus on standardizing PRP protocols and exploring its molecular mechanisms to optimize patient outcomes. Comprehensive guidelines based on robust clinical evidence are needed to integrate PRP effectively into clinical practice for KOA management.

## Figures and Tables

**Table 1:** Benefits and limitations of platelet-rich plasma (PRP) therapy.

Criteria	Benefits	Challenges
Minimally invasive	• No surgery, incisions, or prolonged healing time	• Not applicable
Rapid preparation	• Can be prepared quickly without the need for preservatives	• Not applicable
Safety with own cells	• Uses the patient's own blood, minimizing risks of immune reactions or infections	• Not applicable
Comprehensive therapeutic effects	• Reduces synovial inflammation, protects cartilage, and reduces pain	• Not applicable
Reduction of contaminants	• Closed preparation system minimizes contamination risks	• Not applicable
Accelerated recovery time	• Enhances tissue healing and reduces recovery period	• Not applicable
Enhanced biocompatibility	• Uses the patient's own cells, reducing risk of rejection or immune response	• Not applicable
Morbidity at injection site	• Not applicable	• Possible discomfort, swelling, or pain at the injection site
Lack of standardized methods	• Not applicable	• No universal protocol for PRP preparation or administration, leading to variability in outcomes
Risk of scar tissue and calcification	• Not applicable.	• Potential risk of developing scar tissue or calcification at the injection site
Optimal processing and concentration not fully known	• Not applicable	• Ideal concentration of platelets and growth factors is still under investigation
Risk of infections and allergic reactions	• Not applicable	• Rare risk of infection or allergic reaction, especially if not handled with strict aseptic techniques
Unknown frequency and volume	• Not applicable	• Best frequency and volume of PRP injections for different conditions are not well established
Contraindications for certain conditions	• Not applicable	• Not suitable for patients with specific medical conditions, such as certain autoimmune diseases or cancers, where it could worsen the condition

**Table 2:** Advantages and disadvantages of PRP treatment in TKA.

Outcome parameter	PRP not used	PRP used	Ref.
Pain management	Standard pain levels	Reduced pain levels	[86]
Surgical success	Baseline success rate	Increased success rate	[87]
Blood loss	Moderate reduction	No significant change	
Wound healing	Normal healing process	Accelerated healing	[88]
Postoperative pain control	Conventional management	Enhanced pain control	[89]
Joint range of motion	Better range of motion	Not significantly improved	[86]
Need for postoperative manipulation	Lower rates required	No significant difference	[90]
Joint swelling and circumference	Standard postoperative swelling	Reduced joint swelling	[91]

## Data Availability

All the data processed in this article are part of the research for a doctoral thesis, being archived in the aesthetic medical office, where the interventions were performed.
